# Discovery of Three New Monoterpenoid Indole Alkaloids from the Leaves of *Gardneria multiflora* and Their Vasorelaxant and AChE Inhibitory Activities

**DOI:** 10.3390/molecules26237191

**Published:** 2021-11-27

**Authors:** Sheng-Yuan Zhang, Zi-Wei Li, Jie Xu, Qiu-Ling Chen, Min Song, Qing-Wen Zhang

**Affiliations:** 1 Guangdong Provincial Key Laboratory of Conservation and Precision Utilization of Characteristic Agricultural Resources in Mountainous Areas, Jiaying University, Meizhou 514015, China; mcdullzhang@163.com; 2 NMPA Key Laboratory for Quality Evaluation of TCM, Guangdong Provincial Engineering Research Center for Modernization of TCM, Jinan University, Guangzhou 510632, China; liziweijie@outlook.com (Z.-W.L.); xu908613808@163.com (J.X.); ChenQiuling0926@163.com (Q.-L.C.); 3 State Key Laboratory of Quality Research in Chinese Medicine and Institute of Chinese Medical Sciences, University of Macau, Macau 999078, China; yb77528@um.edu.mo; 4 Department of Pharmaceutical Sciences, Faculty of Health Sciences, University of Macau, Macau 999078, China

**Keywords:** *Gardneria multiflora*, monoterpenoid indole alkaloid, vasorelaxant activity, AChE inhibitory activity

## Abstract

Three novel monoterpenoid indole alkaloids gardflorine A (**1**), gardflorine B (**2**), and gardflorine C (**3**) were isolated from the leaves of *Gardneria multiflora*. Their structures, including absolute configurations, were established on the basis of spectroscopic methods (MS, UV, IR, 1D and 2D NMR) and circular dichroism experiments. All the compounds were evaluated for their vasorelaxant and acetylcholinesterase (AChE) inhibitory activities. Compound **1** exhibited potent vasorelaxant activity, with an EC_50_ value of 8.7 μM, and compounds **2** and **3** showed moderate acetylcholinesterase (AChE) inhibitory activities, with IC_50_ values of 26.8 and 29.2 μM, respectively.

## 1. Introduction

Monoterpenoid indole alkaloids (MIAs) are important secondary metabolites widely distributed in the members of plant families Apocynaceae, Loganiaceae, and Rubiaceae [1]. These compounds have attracted considerable interest in drug research for their complex structures and diverse biological activities, such as ganglion blocking [2], anticancer [3,4,5], anti-inflammatory [6], antibacterial [7], vasorelaxant [8], and neuroprotective activities [9]. The plant *Gardneria multiflora* (Loganiaceae family) is widely distributed in the south of the Qinling Mountains-Huaihe River Line and north of the Nanling Mountains in China [10]. The roots and leaves of *G. multiflora* are widely used as medicine for the treatment of arthrophlogosis and sciatica owing to their effects of expelling wind and activating blood flow [10]. Previous phytochemical investigations of plants from this genus led to the isolation of more than 50 compounds [11,12], most of them were identified as MIAs [13,14,15,16,17].

**Figure 1:**
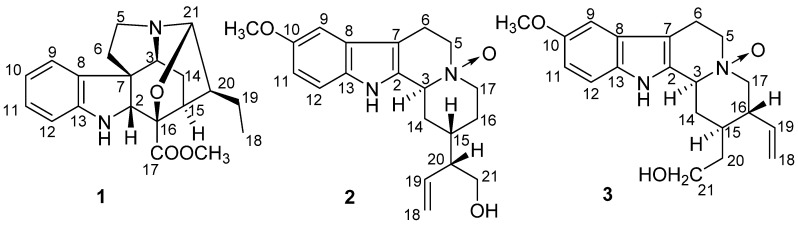
Chemical structures of compounds **1**–**3.**

In search of novel and bioactive alkaloids, we carried out a phytochemical investigation on the constituents of the leaves of *G. multiflora*. In our present study, three new alkaloids (Figure 1), named gardflorine A (**1**), gardflorine B (**2**), and gardflorine C (**3**), were isolated from the leaves of *G. multiflora*. Their structures were elucidated by using spectroscopic methods and electronic circular dichroism (ECD) calculation. Additionally, compound **1** exhibited potent vasorelaxant activity, with an EC_50_ value of 8.7 μM, while, compounds **2** and **3** showed moderate AChE inhibitory activities, with IC_50_ values of 26.8 and 29.2 μM, respectively. Herein, we describe the isolation, structural elucidation, and biological activities of **1**–**3**.

## 2. Results and Discussion

Compound **1** was obtained as white oil, and its molecular formula was established as C_20_H_24_N_2_O_3_ by HR-ESI-MS at *m*/*z* 341.1859 [M + H]^+^ (calcd for C_20_H_25_N_2_O_3_, 341.1860). The IR spectrum of **1** indicated the presence of amino group (3355 cm^−1^), carbonyl group (1736 cm^−1^), and aromatic ring (1609 and 1464 cm^−1^). The ^1^H NMR spectrum of **1** (Table 1) showed signals for an ortho-disubstituted benzene ring [*δ*_H_ 7.08 (1H, dd, *J* = 7.5, 1.3 Hz), 7.02 (1H, td, *J* = 7.5, 1.3 Hz), 6.72 (1H, td, *J* = 7.5, 1.3 Hz), and 6.59 (1H, dd, *J* = 7.5, 1.3 Hz)], a methyl [*δ*_H_ 0.96 (3H, t, *J* = 7.4 Hz)], and a methoxy [*δ*_H_ 3.82 (3H, s)]. The ^13^C NMR and DEPT-135 spectra showed twenty carbon signals, including one carbonyl (*δ*_C_ 174.4), six olefinic carbons (*δ*_C_ 152.7, 133.4, 129.4, 123.6, 120.1, 111.0), two quaternary carbons (*δ*_C_ 85.5, 58.0), five methines (*δ*_C_ 97.5, 73.9, 67.5, 55.0, 39.7), four methylenes (*δ*_C_ 53.2, 44.6, 25.8, 24.5), one methoxy (*δ*_C_ 53.2), and one methyl (*δ*_C_ 11.8). With the aid of 2D NMR spectra, the ^1^H and ^13^C NMR signals of **1** were assigned as shown in Table 1.

**Figure 2:**
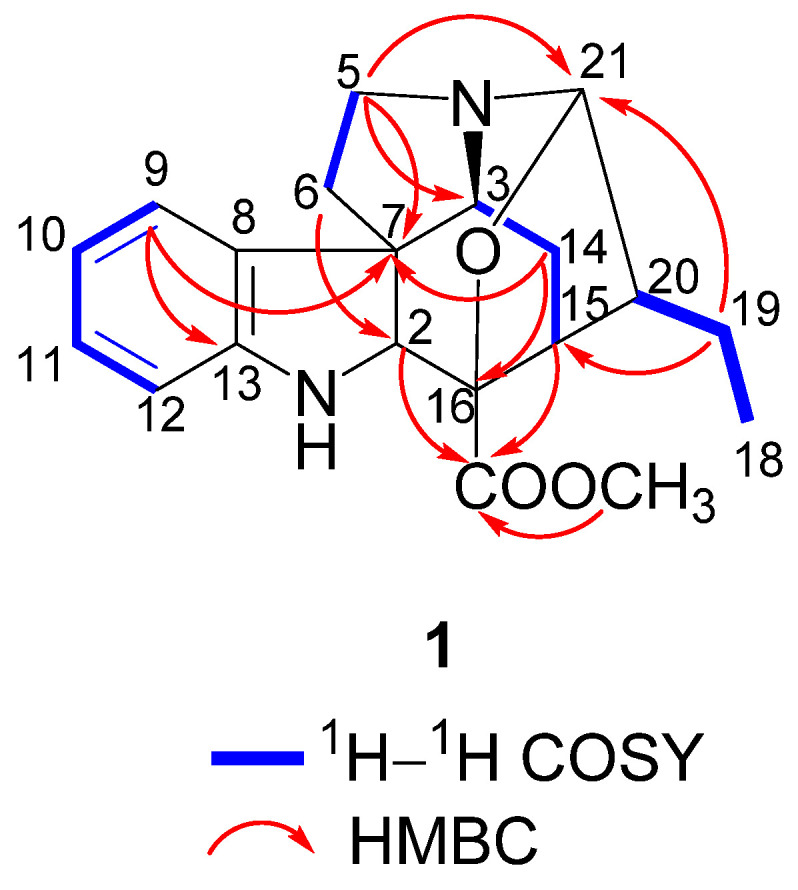
Key ^1^H–^1^H COSY and HMBC correlations of **1**.

The ^1^H–^1^H COSY spectrum revealed the presence of four spin-coupling systems (H-9 to H-12, H_2_-5 to H_2_-6, H-3 to H-15, H_3_-18 to H-20) as shown in Figure 2. The HMBC correlations between H-9 (*δ*_H_ 7.08) and C-7 (*δ*_C_ 58.0)/C-13 (*δ*_C_ 152.7), between H-5*β* (*δ*_H_ 3.17) and C-3 (*δ*_C_ 67.5)/C-7 (*δ*_C_ 58.0)/C-21 (*δ*_C_ 97.5), between H_2_-6 (*δ*_H_ 2.50, 2.29) and C-2 (*δ*_C_ 73.9), between H_2_-19 (*δ*_H_ 1.30, 1.22) and C-15 (*δ*_C_ 39.7)/C-21 (*δ*_C_ 97.5), between H-14*β* (*δ*_H_ 1.78) and C-7 (*δ*_C_ 58.0)/C-16 (*δ*_C_ 85.8), and between H-2 (*δ*_H_ 4.03)/H-15 (*δ*_H_ 2.93) and C-17 (*δ*_C_ 174.4) indicated the presence of the skeleton of akuammicine alkaloid [18]. Subsequently, the HMBC correlation between OCH_3_ (*δ*_H_ 3.82) and C-17 (*δ*_C_ 174.4) suggested that the methoxy connected to C-17. Furthermore, HMBC correlation between H-21 (*δ*_H_ 4.59) and C-16 (*δ*_C_ 85.8), combined with obvious downfield NMR shifts of C-16 (*δ*_C_ 85.8) and C-21 (*δ*_C_ 97.5) and the molecular formula of **1,** suggested C-16 and C-21 were connected via an oxygen atom. Therefore, the planar structure of **1** was established as shown in Figure 2.

**Figure 3:**
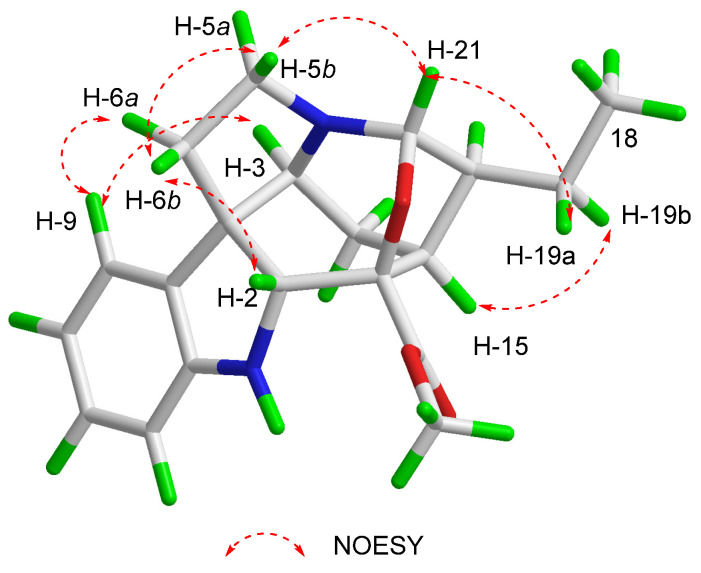
Key NOESY correlations of **1**.

**Figure 4:**
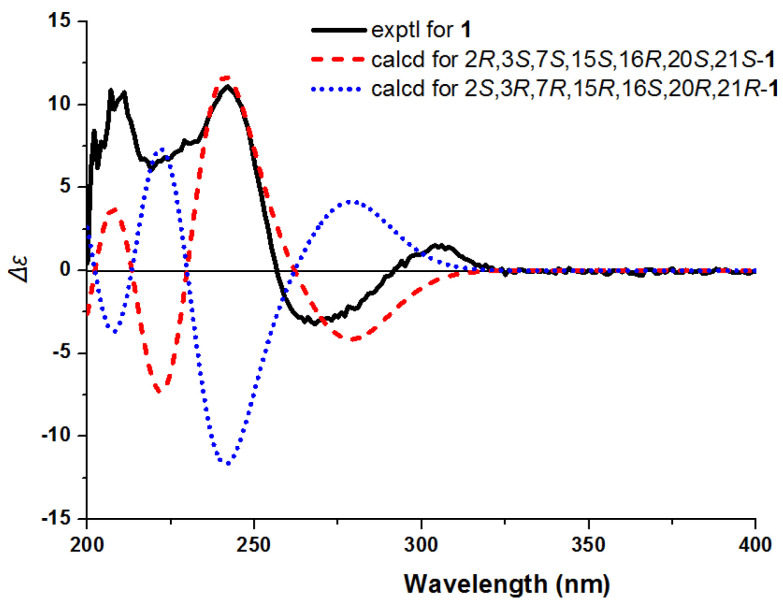
Experimental and calculated ECD spectra of **1**.

The 2D structure of compound **1** determined by the NOESY cross-peaks (Figure 3) between H-9 and H-3/H-6*α*, between H-14*α* and H-3/H-15, and between H-20 and H-15/H-21 indicated the same orientation of these protons. The NOESY cross-peaks between H-6*β* and H-2 indicated that H-2 was *β*-oriented. The absolute configuration of **1** was identified by CD experiment (Figure 4), and the negative Cotton effect at *λ*_max_ 275 (−2.8) nm and the positive Cotton effects at *λ*_max_ 245 (+11.7) nm and 210 (+11.2) nm in CD spectrum were consistent with the calculated configuration of (2*R*, 3*S*, 7*S*, 15*S*, 16*R*, 20*S*, 21*S*)-**1**. Consequently, compound **1** was identified and named as gardflorine A.

**Figure 5:**
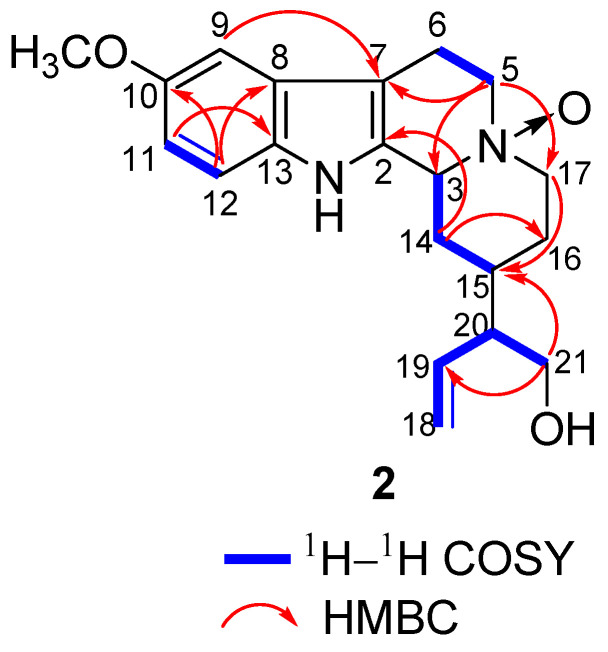
Key ^1^H–^1^H COSY and HMBC correlations of **2**.

**Figure 6:**
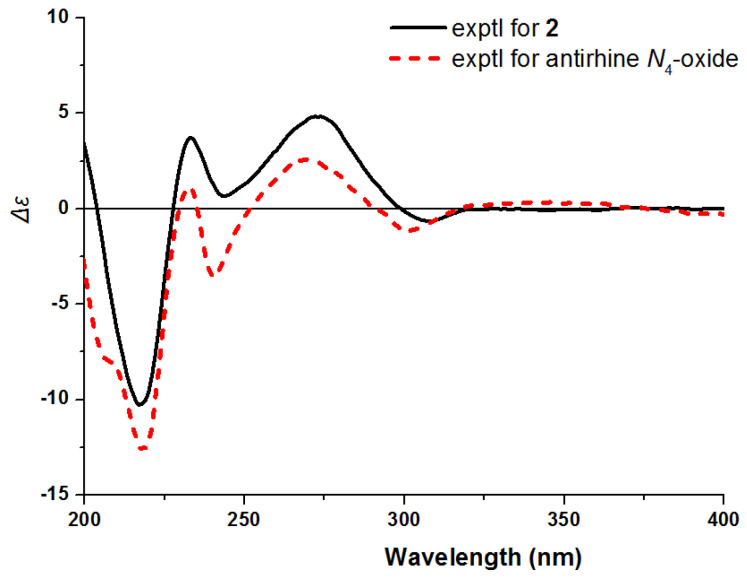
Experimental ECD spectra of **2** and antirhine *N*_4_-oxide.

Compound **2** was isolated as yellow powder, and its molecular formula was established as C_20_H_26_N_2_O_3_ according to HR-ESI-MS at *m*/*z* 343.2020 [M + H]^+^ (calcd for C_20_H_27_N_2_O_3_, 343.2016). The IR spectrum of **2** revealed the presence of amino group (3404 cm^−1^), hydroxy group (3215 cm^−1^) and aromatic ring (1444 cm^−1^). The ^1^H NMR spectrum of **2** (Table 1) showed three aromatic protons [*δ*_H_ 7.24 (1H, d, *J* = 8.8 Hz), 6.93 (1H, d, *J* = 2.4 Hz), and 6.79 (1H, dd, *J* = 8.8, 2.4 Hz)], three olefinic protons [*δ*_H_ 5.69 (1H, d, *J* = 16.9 Hz) and 5.15 (2H, m)], and one methoxy proton [*δ*_H_ 3.80 (3H, s)]. The ^13^C NMR and DEPT-135 spectra showed twenty carbon signals due to ten olefinic carbons (*δ*_C_ 155.6, 138.3, 133.9, 131.4, 128.0, 118.4, 113.3, 113.2, 106.3, 101.0), three methines (*δ*_C_ 71.7, 52.3, 30.7), six methylenes (*δ*_C_ 69.2, 63.9, 59.2, 28.6, 23.6, 20.6), and one methoxy (*δ*_C_ 56.2). Comparison of the NMR data of **2** with those of the known compound antirhine *N*_4_-oxide [19] showed that they were very similar except for the presence of an additional methoxy group in **2**. The chemical shifts of C-10, C-9, C-11, and C-13 shifted from *δ*_C_ 120.6, 119.0, 123.2, and 138.8 in antirhine *N*_4_-oxide to *δ*_C_ 155.6, 101.0, 113.3, and 128.0 in **2**, suggesting that the methoxy group might be connected to C-10. This was confirmed by HMBC correlations from *δ*_H_ 3.80 (OCH_3_) to *δ*_C_ 155.6 (C-10) (Figure 5). The absolute configuration of **2** was determined by comparing the ECD spectrum of **2** with that of antirhine *N*_4_-oxide (Figure 6). Therefore, compound **2** was identified as 10-methoxyantirhine *N*_4_-oxide and named as gardflorine B.

**Figure 7:**
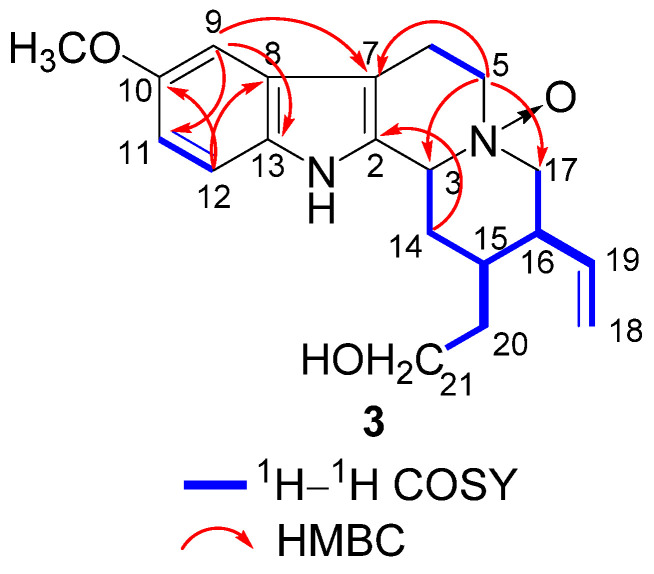
Key ^1^H–^1^H COSY and HMBC correlations of **3**.

**Figure 8:**
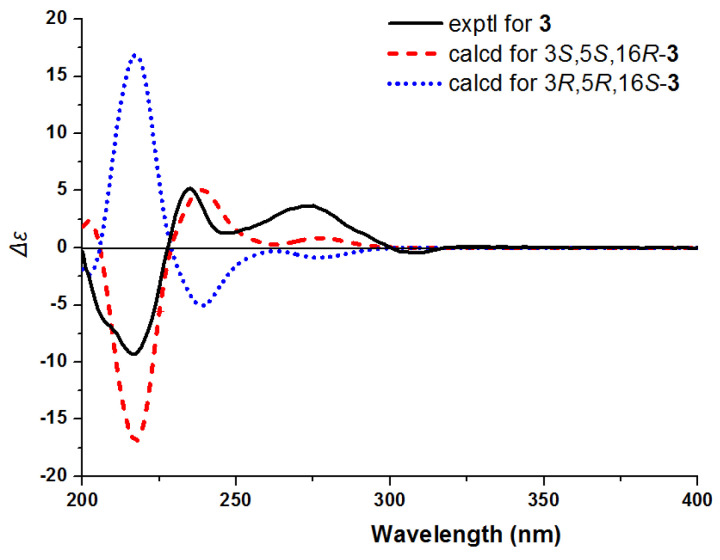
Experimental and calculated ECD spectra of **3**.

The molecular formula of **3** was established as C_20_H_26_N_2_O_3_ according to an [M + H]^+^ ion peak at *m*/*z* 343.2012 [M + H]^+^ (calcd for C_20_H_27_N_2_O_3_, 343.2016) in the HR-ESI-MS spectrum. Its IR spectrum suggested the presence of amino group (3396 cm^−1^), hydroxy group (3230 cm^−1^), and aromatic ring (1461 cm^−1^). The ^1^H and ^13^C NMR spectrum of **3** (Table 1) showed the presence of a 1,2,4-trisubstituted benzene ring [*δ*_H_ 7.20 (1H, d, *J* = 8.2 Hz), 6.91 (1H, d, *J* = 2.0 Hz), 6.79 (1H, dd, *J* = 8.2, 2.0 Hz); *δ*_C_ 155.6, 133.9, 128.0, 113.3, 113.2, 101.0], a terminal double bond [*δ*_H_ 5.65 (1H, m), 5.11 (2H, m); *δ*_C_ 138.3, 118.5], and one methoxy signal [*δ*_H_ 3.78 (3H, s); *δ*_C_ 56.2]. The 1D NMR data of **3** (Table 1) closely resembled to those of **2**; however, their 2D NMR data showed many differences. The ^1^H–^1^H COSY correlations of H-3 (*δ*_H_ 4.60)/H_2_-14 (*δ*_H_ 2.53, 2.23)/H-15 (*δ*_H_ 1.49)/H-16 (*δ*_H_ 2.13)/H_2_-17 (*δ*_H_ 3.51, 3.01), H-15/H_2_-20 (*δ*_H_ 2.03, 1.49)/H_2_-21 (*δ*_H_ 3.58, 3.51), and H-16/H-19 (*δ*_H_ 5.65)/H_2_-18 (*δ*_H_ 5.11), together with the HMBC correlations between H_2_-5 (*δ*_H_ 3.69) and C-17 (*δ*_C_ 59.1), between H_2_-17 and C-19 (*δ*_C_ 138.3), between H_2_-18 and C-16 (*δ*_C_ 52.3), and between H_2_-21 and C-15 (*δ*_C_ 30.6) revealed that the terminal double bond was linked to C-16 (Figure 7). The NOESY correlations between H-15 and H-3/H-19 established the relative configuration of **3**. Subsequently, the absolute configuration of **3** was identified by CD experiment (Figure 8). The positive Cotton effect at *λ*_max_ 272 (+3.7) nm and 235 (+5.2) nm and the negative Cotton effect at *λ*_max_ 217 (−9.3) nm displayed good agreement with the calculated ECD curve for (3*S*, 15*S*, 16*R*)-**3**. Consequently, compound **3** was identified as 10-methoxycorynantheol *N*_4_-oxide and named as gardflorine C.

In order to explore the scientific connotation of the traditional use of *G. multiflora*, the vasorelaxant and AChE inhibitory activities in vitro of compounds **1**–**3** were evaluated. Among them, compound **1** exhibited potent vasorelaxant activity, with an EC_50_ value of 8.7 μM (EC_50_ = 0.1 μM for positive control phentolamine mesylate). Moreover, compounds **2** and **3** exhibited moderate AChE inhibitory activities, with IC_50_ values of 26.8 and 29.2 μM, respectively (Supplementary Materials).

## 3. Materials and Methods

### 3.1. General Experimental Procedures

UV and IR spectra were obtained on a JASCO V-550 spectrophotometer and a JASCO FI/IR-480 Plus Fourier transform infrared spectrometer, respectively. CD spectra were recorded on a Chirascan spectropolarimeter. Optical rotations were determined with a JASCO P-1020 Automatic Polarimeter. HR-ESI-MS data were obtained using an Agilent 6210 ESI/TOF mass spectrometer. NMR experiments were performed on Bruker AV-600 and AV-400 spectrometers. HPLC was carried out on an Agilent 1260 chromatograph and a semi-preparative chromatograph with a DAD detector. 

Column chromatography (CC) was performed on silica gel (60–80 mesh, 200–300 mesh, Qingdao Marine Chemical Inc., Qingdao, China), Sephadex LH-20 (Pharmacia Biotech AB), and YMC-Pack ODS (Merck). TLC was carried out on glass precoated silica gel GF_254_ plates. Waters X-bridge C_18_ column (250 × 4.6 mm, 5 μm; 250 × 10 mm, 5 μm) was used to analyze and isolate the compounds.

### 3.2. Plant Materials

The leaves of *Gardneria multiflora* were collected in Yangchang Town, Longli County, Guizhou province, in June 2018 and identified by Dr. Ying Zhang of Jinan University. A voucher specimen has been deposited at the Medical College of Jiaying University (No. MCJU-021).

### 3.3. Extraction and Isolation

The dried, powdered leaves of *G. multiflora* (20 kg) were percolated with 95% ethanol (100 L×3). After evaporation of solvent in vacuum, the residue (1.5 kg) was suspended in water, and the pH was adjusted to 2–3 by 5% HCl and then partitioned with chloroform; thus, the chloroform layer and acid water layer were obtained. The acid water layer was then adjusted to pH 9–10 by ammonia water, chloroform extraction was carried out, and the crude total alkaloid (chloroform part) was obtained. The chloroform extract (31.3 g) was subjected to a silica gel column eluting with chloroform/methanol (100:0 to 0:100, *v/v*) to afford 8 fractions (A1–A8). Then, fraction A4 was further separated by a silica gel, ODS, Sephadex LH-20 columns, and preparative HPLC to afford **1** (2.7 mg). Fraction A6 was successively separated on Sephadex LH-20 (MeOH) a20nd purified by preparative HPLC with MeOH–H_2_O–Et_2_NH (60:40:0.0002) to afford **2** (6.6 mg) and **3** (5.9 mg).

### 3.4. Spectral Data

#### 3.4.1. Gardflorine A (1)

White oil, [*α*${]}_{D}^{25}$ +36.1 (*c* 0.92, MeOH); UV(MeOH) *λ*_max_ (log ε) 209 (4.31), 245 (3.22), 299 (4.05); IR (KBr) *ν*_max_ 3355, 2957, 2877, 1736, 1674, 1609, 1485, 1464, 1383, 1308, 750 cm^−1^; HR-ESI-MS *m*/*z* 341.1859 [M + H]^+^ (calcd for C_20_H_25_N_2_O_3_, 341.1860); ^1^H and ^13^C NMR data, see Table 1.

#### 3.4.2. Gardflorine B (2)

Yellow powder, [*α*${]}_{D}^{25}$ +49.0 (*c* 1.01, MeOH); UV(MeOH) *λ*_max_ (log ε) 211 (4.33), 275 (3.81); IR (KBr) *ν*_max_ 3404, 3215, 2929, 1622, 1444, 1327, 1227, 1115, 742, 630 cm^−1^; HR-ESI-MS *m*/*z* 343.2020 [M + H]^+^ (calcd for C_20_H_27_N_2_O_3_, 343.2016); ^1^H and ^13^C NMR data, see Table 1.

#### 3.4.3. Gardflorine C (3)

Yellow powder, [*α*${]}_{D}^{25}$ −17.4 (*c* 0.70, MeOH); UV(MeOH) *λ*_max_ (log ε) 206 (5.02), 274 (4.45); IR (KBr) *ν*_max_ 3396, 3230, 3056, 2925, 1638, 1461, 1330, 1234, 1156, 1079, 742, 512 cm^−1^; HR-ESI-MS *m*/*z* 343.2012 [M + H]^+^ (calcd for C_20_H_27_N_2_O_3_, 343.2016); ^1^H and ^13^C NMR data, see Table 1.

### 3.5. Vasorelaxant Assay

The vasorelaxant activity of these isolates against KCl-induced contractions of rat renal artery rings was measured as described previously [20,21,22]. Renal arteries were removed rapidly out from SD rats, immediately placed into 4 °C oxygenated K-H solution, cleaned of its surrounding fat and connective tissues, and then cut into portions of about 2 mm in length. Each segment was mounted in a Multi Myograph System (Danish Myo Technology A/S, Denmark) and then bathed in K-H solution [composition (in mM): NaCl, 120; KCl, 4.6; KH_2_PO_4_, 1.2; MgSO_4_, 1.2; NaHCO_3_, 25; glucose, 10; CaCl_2_, 2.5], bubbled with 95% O_2_–5% CO_2_, and maintained at 37 °C. The isometric tension of renal artery rings was collected by four-channel physiological force transducers. All the rings were set to an optimal tension of 2 g and stabilized in normal K-H solution for 90 min. The rings were then contracted by 0.5 μM phenylephrine and challenged with 3 μM acetylcholine to confirm the integrity of the endothelium. Endothelium-intact rings contraction was evoked by a depolarizing KCl (60 mM) solution. The EC_50_ values of the test compounds and the positive control (phentolamine mesylate) were calculated from cumulative concentration–tension curves by linear regression.

### 3.6. AChE Inhibitory Activity Assay

The AChE inhibitory activities of the isolated compounds were assayed by a modified Ellman’s method [6,23]. Compounds and positive control were dissolved in 1% DMSO. The phosphate buffer (pH 8.0), tacrine, test compounds, and acetylcholinesterase (0.02 μM) were added, in sequence to 96-well plates and incubated for 20 min (30 °C). The reaction was initiated by the addition of 20 μL of 5,5′-dithiobis-(2-nitrobenzoic acid) (DTNB) (0.625 mM) and 20 μL of acetylthiocholine iodide (0.625 mM) for the AChE inhibitory activity assay, respectively. The optical density was measured at 405 nm by an ELISA microplate reader. Tacrine (IC_50_ 0.33 μM) was used as positive control. All the reactions were performed in triplicate. The percentage inhibition (I%) was calculated as follows: I% = (1 − S)/E × 100 (S is the absorbance of the test compound-containing reaction, and E is the absorbance of the control reaction).

## 4. Conclusions

In summary, three new monoterpenoid indole alkaloids (**1**–**3**) were isolated and identified from the leaves of *G. multiflora*. The new compounds were elucidated by spectroscopic analyses and computational calculation. Moreover, the vasorelaxant and AChE inhibitory activities of all isolates were tested. Compound **1** exhibited potent vasorelaxant activity, with an EC_50_ value of 8.7 μM, while compounds **2** and **3** exhibited AChE inhibitory activities, with IC_50_ values of 26.8 and 29.2 μM, respectively. The discovery of the new alkaloids expands the family of MIAs and provides reference for further structure–activity discussions in future research.

## Figures and Tables

**Table 1:** NMR data of 1–3 (CD_3_OD, *δ* in ppm, *J* in Hz).

Position		1 *^a^*			2 *^a^*			3 *^b^*	
		*δ*_H_	*δ*_C_, Type		*δ*_H_	*δ*_C_, Type		*δ*_H_	*δ*_C_, Type
2		4.03, s	73.9, CH		-	131.4, C		-	131.4, C
3		2.89, d (4.7)	67.5, CH		4.61, s	71.7, CH		4.60, brs	71.6, CH
5	*α* *β*	3.30, m 3.17, m	53.2, CH_2_	a/b	3.72, dd (9.2, 4.0)	69.2, CH_2_	a/b	3.69, m	69.2, CH_2_
6	*α* *β*	2.50, m 2.29, m	44.6, CH_2_	a/b	3.06, m	20.6, CH_2_	*α* *β*	3.07, m 2.98, m	20.6, CH_2_
7		-	58.0, C		-	106.3, C		-	106.2, C
8		-	133.4, C		-	128.0, C		-	128.0, C
9		7.08, dd (7.5, 1.3)	123.6, CH		6.93, d (2.4)	101.0, CH		6.91, d (2.0)	101.0, CH
10		6.72, td (7.5, 1.3)	120.1, CH		-	155.6, C		-	155.6, C
11		7.02, td (7.5, 1.3)	129.4, CH		6.78, dd (8.8,2.4)	113.3, CH		6.79, dd (8.2,2.0)	113.3, CH
12		6.59, dd (7.5, 1.3)	111.0, CH		7.24, d (8.8)	113.2, CH		7.20, d (8.2)	113.2, CH
13		-	152.7, C		-	133.9, C		-	133.9, C
14	*α* *β*	2.12, m 1.78, m	25.8, CH_2_	*α* *β*	2.58, td (13.6,4.9) 2.25, d (13.6)	28.6, CH_2_	*α* *β*	2.53, m 2.23, m	28.4, CH_2_
15		2.93, d (4.7)	39.7, CH		1.54, overlap	30.7, CH		1.49, overlap	30.6, CH
16		-	85.8, C	*β* *α*	2.07, dd (13.4,3.7)1.54, overlap	23.6, CH_2_		2.13, m	52.3, CH
17		-	174.4, C	*α* *β*	3.57, m 3.05, m	59.2, CH_2_	*α* *β*	3.51, overlap3.01, m	59.1, CH_2_
18		0.96, t (7.4)	11.8, CH_3_		5.15, m	118.4, CH_2_		5.11, m	118.5, CH_2_
19	a b	1.30, m 1.22, m	24.5, CH_2_		5.69, d (16.9)	138.3, CH		5.65, m	138.3, CH
20		1.83, t (7.6)	55.0, CH		2.17, m	52.3, CH	a b	2.03, m 1.49, overlap	23.6, CH_2_
21		4.59, s	97.5, CH		3.59, m	63.9, CH_2_	a b	3.58, m 3.51, overlap	63.8, CH_2_
OCH_3_		3.82, s	53.2, CH_3_		3.80, s	56.2, CH_3_		3.78, s	56.2, CH_3_

*^a^*^1^H NMR spectra of **1** and **2** were recorded at 600 MHz and ^13^C NMR was recorded at 150MHz. *^b^* ^1^H NMR spectrum of **3** was recorded at 400 MHz and ^13^C NMR was recorded at 100 MHz.

## Data Availability

The data of the NMR and vasorelaxant and AchE inhibitory activity presented in this study are available in Supplementary Materials.

## Supplementary Materials

The following are available online. Figure S1: Leaves of *G. multiflora*, Tables S1 and S2: biological activities of compounds **1**–**3**, Figures S2–S10: HR-ESI-MS, UV, IR, 1D and 2D NMR spectra of compound **1**, Figures S11–S19: HR-ESI-MS, UV, IR, 1D and 2D NMR spectra of compound **2**, Figures S20–S28: HR-ESI-MS, UV, IR, 1D and 2D NMR spectra of compound **3**.

## Sample Availability

Samples of the compounds **1**–**3** are available from the authors.

## References

[1] Zhang J., Yuan M.F., Li S.T., Sang C.C., Chen M.F., Ao Y.L., Li Z.W., Xie J., Ye W.C., Zhang X.Q. (2020). Hunzeylanines A–E, five bisindole alkaloids tethered with a methylene group from the roots of *Hunteria zeylanica*. Org. Chem..

[2] Masatoshi H., Yukihiro O. (1978). Effect of gardneria alkaloids on ganglionic transmission in the rabbit and rat superior cervical ganglia in situ. Chem. Pharm. Bull..

[3] Harada M., Ozaki Y. (1976). Effect of indole alkaloids from *Gardneria* genus and Uncaria genus on neuromuscular transmission in the rat limb in situ. Chem. Pharm. Bull..

[4] Feng T., Li X.L., Zhang B.H., Li Y., Cai X.H., Liu Y.P., Luo X.D. (2013). Gardovatine, a novel Strychnos-Strychnos bisindole alkaloid with cytotoxicity from *Gardneria oveta*. Bioorg. Med. Chem. Lett..

[5] Yan H.S., Yan H.D. (2019). Research advance on Dai medicine *Gardneria multiflora*. J. Med. Pharm.Chin. Minorities..

[6] Zhang W., Xu W., Wang G.Y., Gong X.Y., Li N.P., Wang L., Ye W.C. (2017). Gelsekoumidines A and B: two pairs of atropisomeric bisindole alkaloids from the roots of *Gelsemium elegans*. Org. Lett..

[7] Ding C.F., Ma H.X., Yang J., Qin X.J., Njateng G.S.S., Yu H.F., Wei X., Liu Y.P., Huang W.Y., Yang Z.F. (2018). Antibacterial indole alkaloids with complex heterocycles from *Voacanga africana*. Org. Lett..

[8] Zhang J., Song M., Ao Y.L., Li Y., Zou X.Y., Xu J., Wang Y., Zhang D.M., Zhang X.Q., Ye W.C. (2020). Alstolarines A and B, two unusual monoterpenoid indole alkaloids with acetal moiety from *Alstonia scholaris*. Org. Chem. Front..

[9] He Q.F., Wu Z.L., Li L., Sun W.Y., Wang G.Y., Jiang R.W., Hu L.J., Shi L., He R.R., Wang Y. (2021). Discovery of neuritogenic securinega alkaloids from *Flueggea suffruticosa* by a building blocks-based molecular network strategy. Angew. Chem. Int. Ed..

[10] Editorial Committee of Flora of China (1992). Flora of China.

[11] Xie G.H., Ma L., Zheng Z.P., Hu L.H. (2007). Lignans from *Gardneria multiflor*. Chin. J. Nat. Med..

[12] Akayama H., Nitta W., Kitajima M., Aimi N., Sakai S. (1994). A new Gardneria alkaloid, gardquinolone, having a novel 4-quinolone skeleton. J. Nat. Prod..

[13] Li X.N., Cai X.H., Feng T., Li Y., Liu Y.P., Luo X.D. (2011). Monoterpenoid Indole Alkaloids from *Gardneria ovata*. J. Nat. Prod..

[14] Zhong X.H., Xiao L., Wang Q., Zhang B.J., Bao M.F., Cai X.H., Peng L. (2014). Cytotoxic 7*S*-oxindole alkaloids from *Gardneria multiflora*. Phytochem. Lett..

[15] Yang W.X., Huang T., Zhang J.X., Liu J.H., Hao X.J., Zhang Y.H. (2016). A New Monoterpenoid Indole Alkaloid from *Gardneria multiflora* Makino. Chin. Pharm. J..

[16] Yang W.X., Chen Y.F., Yang J., Huang T., Wu L.L., Xiao N., Hao X.J., Zhang Y.H. (2018). Monoterpenoid indole alkaloids from *Gardneria multiflora*. Fitoterapia.

[17] Si Y.Y., Wang W.W., Feng Q.M., Zhao Z.Z., Xue G.M., Sun Y.J., Feng W.S., Young J.I., Wang X.S. (2021). Neuroinflammatory inhibitors from *Gardneria nutans* Siebold & Zuccarini. RSC Adv..

[18] Gan L.S., Yang S.P., Wu Y., Ding J., Yue J.M. (2006). Terpenoid Indole Alkaloids from *Winchia calophylla*. J. Nat. Prod..

[19] Jiang H., Liu Y.B., Li Y., Li L., Ma S.G., Qu J., Yu S.S. (2016). Analgesic corynanthe-type alkaloids from *Strychnos angustiflora*. Tetrahedron.

[20] Hu G.Y., Li X.X., Zhang S.Y., Wang X. (2016). Association of rat thoracic aorta dilatation by astragaloside IV with the generation of endothelium-derived hyperpolarizing factors and nitric oxide, and the blockade of Ca^2+^ channels. Biomed. Rep..

[21] Xie J., Zou X.Y., Sang C.C., Song M., Chen Q.L., Zhang J. (2021). Three new monoterpenoid indole alkaloids from *Alstonia rostrata*. Tetrahedron Lett..

[22] Zhang J., Liu Z.W., Li Y., Wei C.J., Xie J., Yuan M.F., Zhang D.M., Ye W.C., Zhang X.Q. (2020). Structurally Diverse Indole Alkaloids with Vasorelaxant Activity from *Melodinus hemsleyanus*. J. Nat. Prod..

[23] Lou H.Y., Yi P., Hu Z.X., Li Y.N., Zeng Y.R., Gu W., Huang L.J., Yuan C.M., Hao X.J. (2020). Polycyclic polyprenylated acylphloroglucinols with acetylcholinesterase inhibitory activities from *Hypericum perforatum*. Fitoterapia.

