# Supplementary material for: Discovery of Three New Monoterpenoid Indole Alkaloids from the Leaves of Gardneria multiflora and Their Vasorelaxant and AChE Inhibitory Activities
Source: Molecules. 2021 Nov 27;26(23):7191. doi: 10.3390/molecules26237191 (PMC8659093; doi:10.3390/molecules26237191)
Supplement: Supplementary file 1 [file molecules-26-07191-s001.zip › molecules-1474821-supplementary.pdf]

Supporting Information

Discovery of three new monoterpenoid indole alkaloids from the leaves of *Gardneria multiflora* and their vasorelaxant and AChE inhibitory activities

Sheng-Yuan Zhang[1,†], *Zi-Wei Li*[2,†], *Jie Xu*[2], *Qiu-Ling Chen*[2], *Min Song*[3], *Qing-Wen Zhang*[3,4,*]

[1] Guangdong Provincial Key Laboratory of Conservation and Precision Utilization of Characteristic Agricultural Resources in Mountainous Areas, Jiaying University, Meizhou 514015, P. R China

[2] NMPA Key Laboratory for Quality Evaluation of TCM, and Guangdong Provincial Engineering Research Center for Modernization of TCM, Jinan University, Guangzhou 510632, P. R China

[3] State Key Laboratory of Quality Research in Chinese Medicine and Institute of Chinese Medical Sciences, University of Macau, Macao SAR, P.R. China

[4] Department of Pharmaceutical Sciences, Faculty of Health Sciences, University of Macau, Macao SAR, P. R. China

[*] Correspondence: qwzhang@um.edu.mo (Q. Z.)

[†] These authors contributed equally to this work.

Supporting Information Content:

Fig.S1. The leaves of *Gardneria multiflora*

S3–4. Computational calculation of **1** and **3**

Tab.S1. Vasorelaxant activity of compound **1–3**.

Tab.S2. Acetylcholinesterase (AChE) inhibitory activity of compound **1–3**.

Fig.S2. The UV spectrum of compound **1** (methanol).

Fig.S3. The IR spectrum of compound **1**.

Fig.S4. The HR-ESI-MS spectrum of compound **1**.

Fig.S5-7. The 1D spectra of compound **1** (CD_3OD).

Fig.S8-11. The 2D spectra of compound **1** (CD_3OD).

Fig.S12. The UV spectrum of compound **2** (methanol).

Fig.S13. The IR spectrum of compound **2**.

Fig.S14. The HR-ESI-MS spectrum of compound **2**.

Fig.S15-17. The 1D spectra of compound **2** (CD_3OD).

Fig.S18-21. The 2D spectra of compound **2** (CD_3OD).

Fig.S22. The UV spectrum of compound **3** (methanol).

Fig.S23. The IR spectrum of compound **3**.

Fig.S24. The HR-ESI-MS spectrum of compound **3**.

Fig.S25-27. The 1D spectra of compound **3** (CD_3OD).

Fig.S28-31. The 2D spectra of compound **3** (CD_3OD).

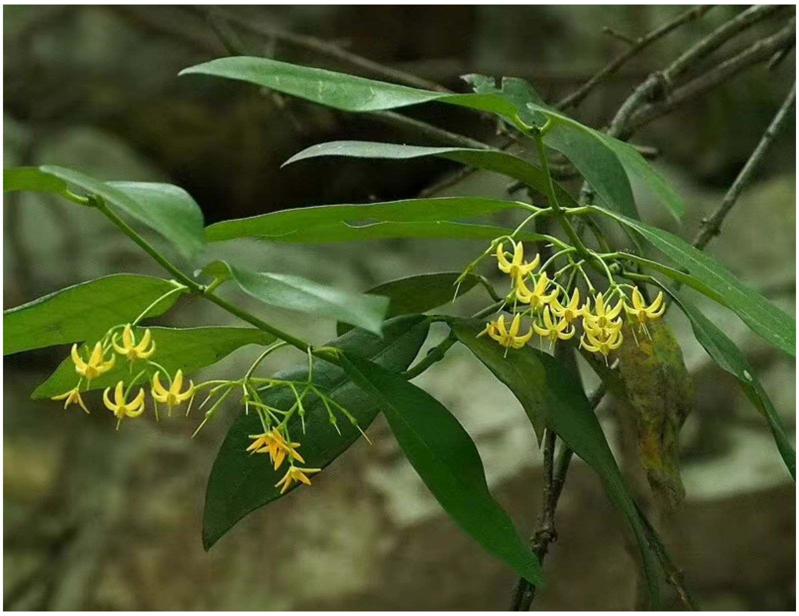

Fig.S1 The leaves of *Gardneria multiflora*

Computational Calculation of 1 and 3

The conformational analysis of compound 1 was performed in the Sybyl 8.1 software by using the MMFF94S molecular force field, which afforded 12 and 46 conformers for **1** and **3**, with an energy cutoff of 10 kcal/mol to the global minima. All the obtained conformers were further optimized using DFT at the B3LYP/6-31+G(d) level in acetonitrile by using Gaussian09 software [1], while 4 and 3 conformers of **1** and **3** were selected. The overall ECD curves of **1** and **3** were weighted by Boltzmann distribution of each conformer (with a half-bandwidth of 0.26eV). The calculated ECD spectra of **1** and **3** were subsequently compared with the experimental ones. The ECD curves were produced by SpecDis 1.6 software [2].

[1] Gaussian 09, Revision A.02, M. J. Frisch, G. W. Trucks, H. B. Schlegel, G. E. Scuseria, M. A. Robb, J. R. Cheeseman, G. Scalmani, V. Barone, B. Mennucci, G. A. Petersson, H. Nakatsuji, M. Caricato, X. Li, H. P. Hratchian, A. F. Izmaylov, J. Bloino, G. Zheng, J. L. Sonnenberg, M. Hada, M. Ehara, K. Toyota, R. Fukuda, J. Hasegawa, M. Ishida, T. Nakajima, Y. Honda, O. Kitao, H. Nakai, T. Vreven, J. A. Montgomery, Jr., J. E. Peralta, F. Ogliaro, M. Bearpark, J. J. Heyd, E. Brothers, K. N. Kudin, V. N. Staroverov, R. Kobayashi, J. Normand, K. Raghavachari, A. Rendell, J. C. Burant, S. S. Iyengar, J. Tomasi, M. Cossi, N. Rega, J. M. Millam, M. Klene, J. E. Knox, J. B.

Cross, V. Bakken, C. Adamo, J. Jaramillo, R. Gomperts, R. E. Stratmann, O. Yazyev, A. J. Austin, R. Cammi, C. Pomelli, J. W. Ochterski, R. L. Martin, K. Morokuma, V. G. Zakrzewski, G. A. Voth, P. Salvador, J. J. Dannenberg, S. Dapprich, A. D. Daniels, O. Farkas, J. B. Foresman, J. V. Ortiz, J. Cioslowski, and D. J. Fox, Gaussian, Inc., Wallingford CT, 2009.

[2] T. Bruhn, A. Schaumlöffel, Y. Hemberger, G. Bringmann, SpecDis version 1.60, University of Wuerzburg, Germany, 2012.

Tab. S1 Vasorelaxant activity of compound **1–3**.

compounds	EC_{50} (μM)
**1**	8.7
**2**	>50
**3**	>50
Phentolamine mesylate	0.2

Tab. S2 Acetylcholinesterase (AChE) inhibitory activity of compound **1–3**.

compounds	IC_{50} (μM)
**1**	>50
**2**	26.8
**3**	29.2
Tacrine	0.33

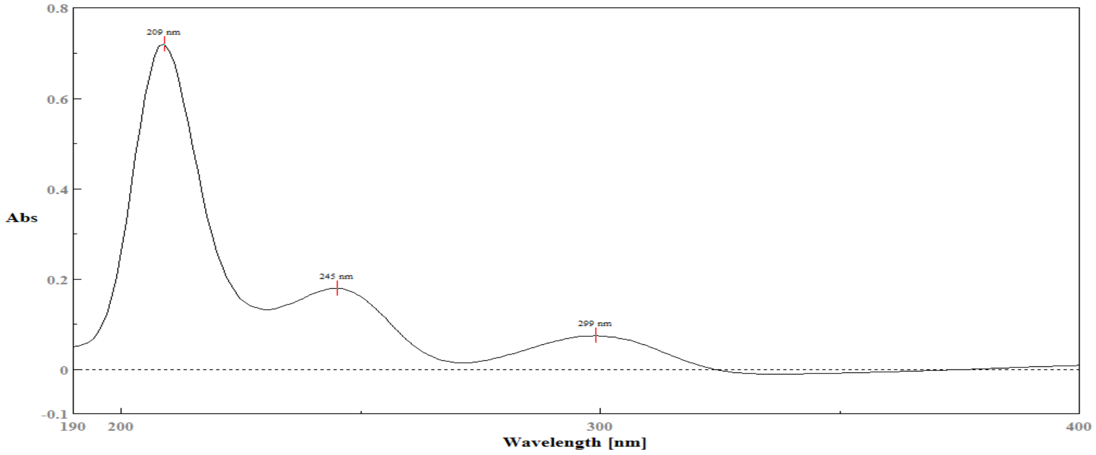

Fig.S2 UV spectrum of **1** (CH_3OH)

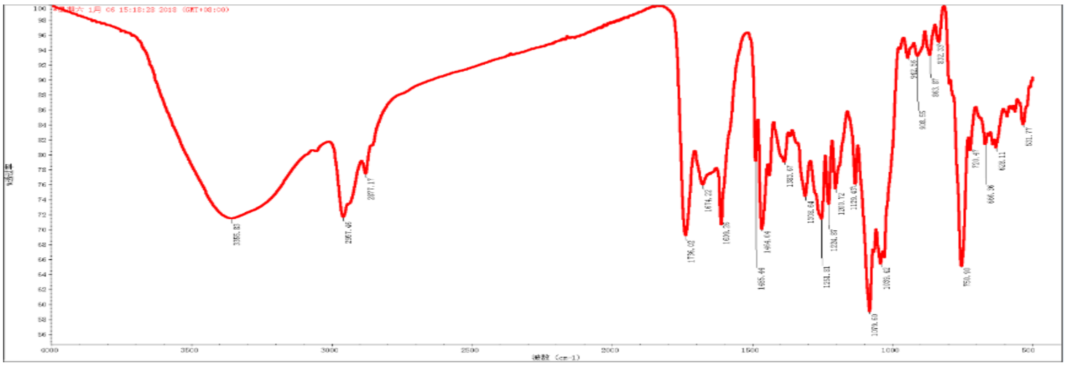

Fig.S3 IR spectrum of **1** (KBr disc)

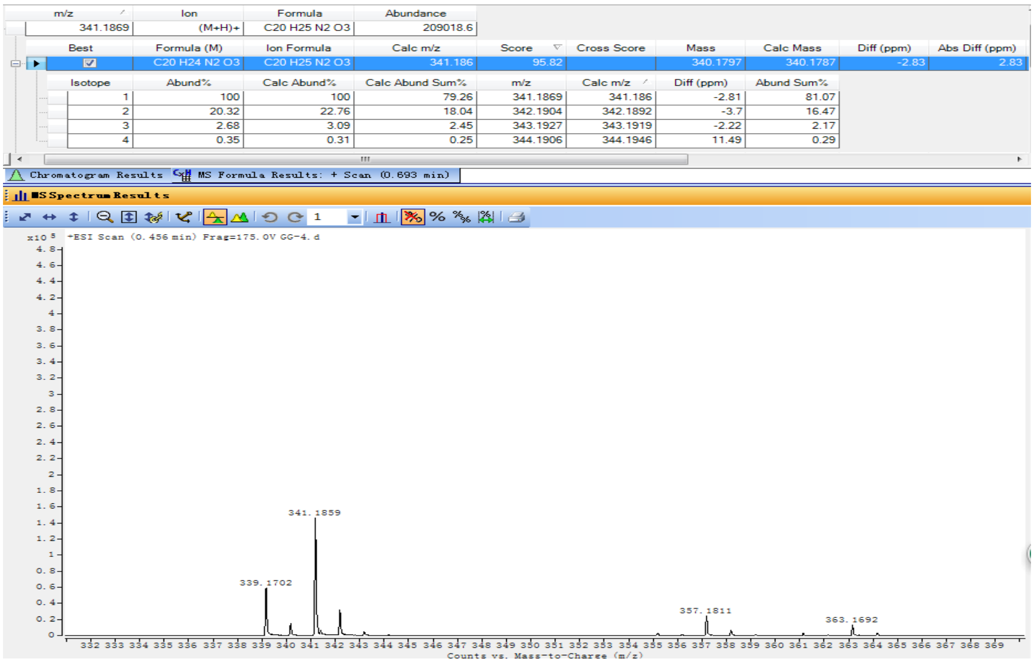

Fig.S4 HR-ESI-MS spectrum of **1**

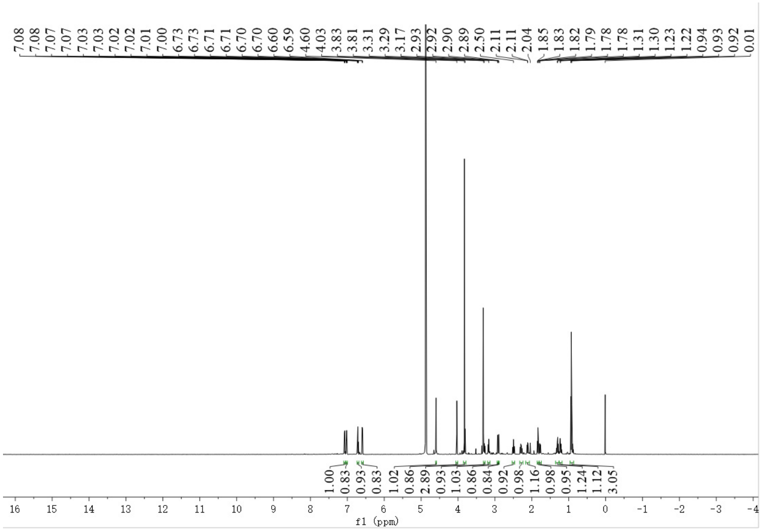

Fig.S5 ^{1}H NMR spectrum of **1** (600 MHz, CD_3OD)

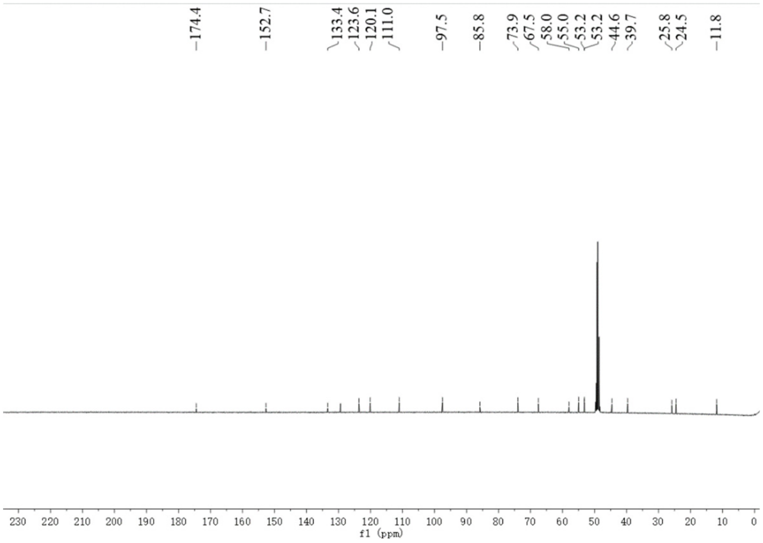

Fig.S6 ^{13}C NMR spectrum of **1** (150 MHz, CD_3OD)

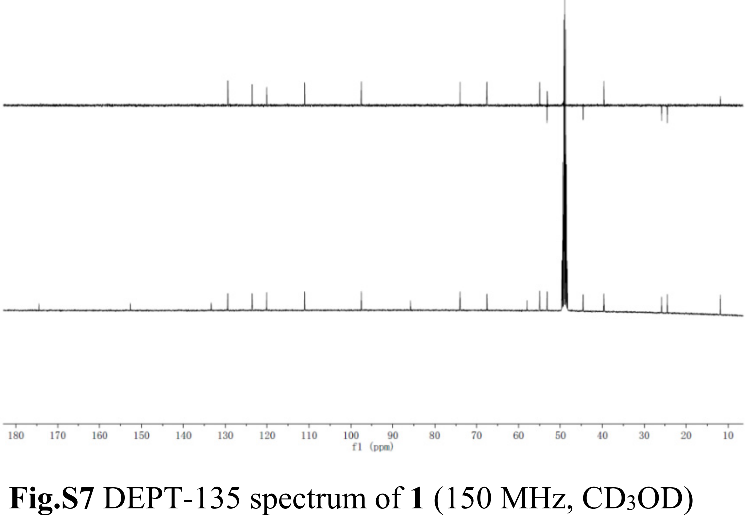

Fig.S7 DEPT-135 spectrum of **1** (150 MHz, CD_3OD)

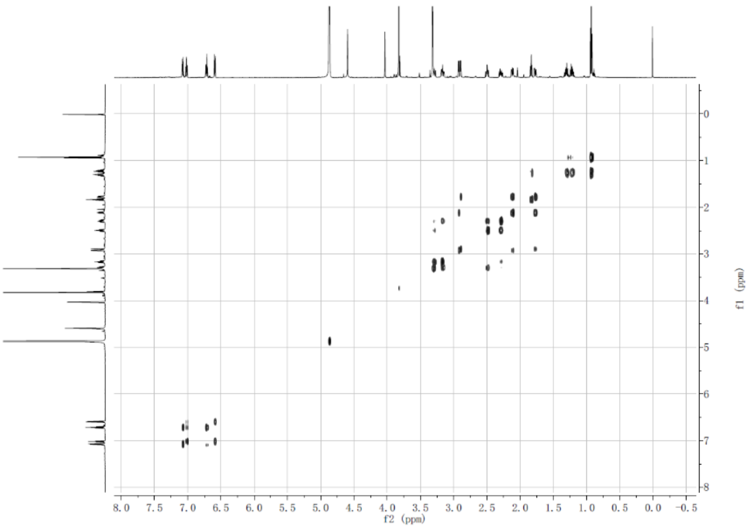

Fig.S8 ^{1}H-^{1}H COSY spectrum of **1**(CD_3OD)

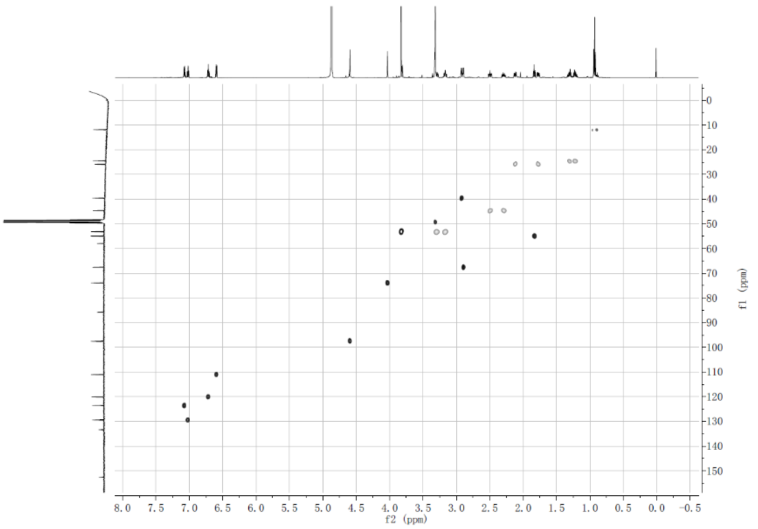

Fig.S9 HSQC spectrum of **1** (CD_3OD)

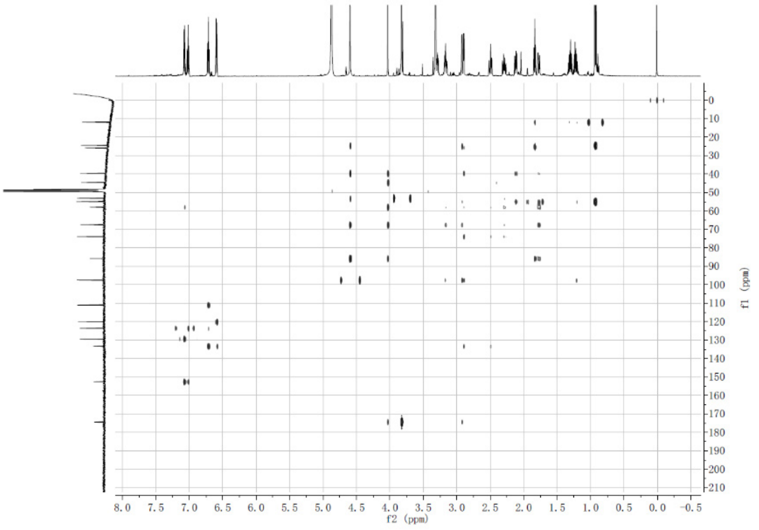

Fig.S10 HMBC spectrum of **1** (CD_3OD)

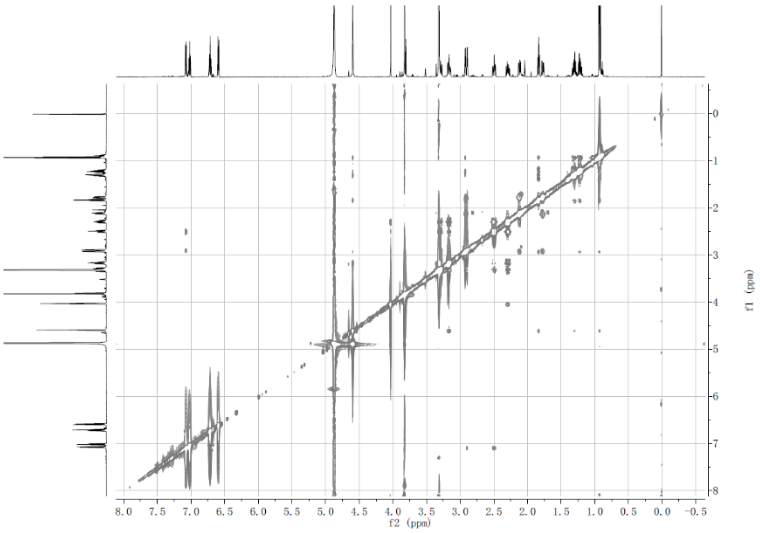

Fig.S11 NOESY spectrum of **1** (CD_3OD)

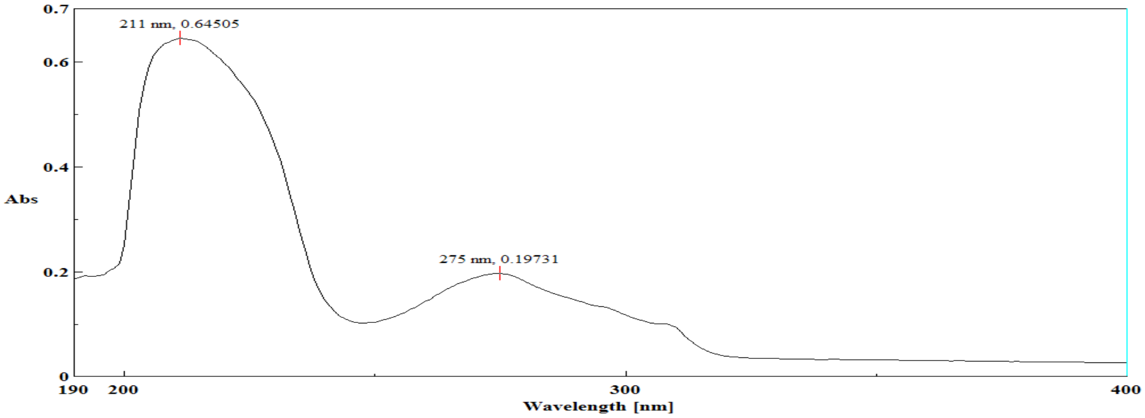

Fig.S12 UV spectrum of **2** (CH_3OH)

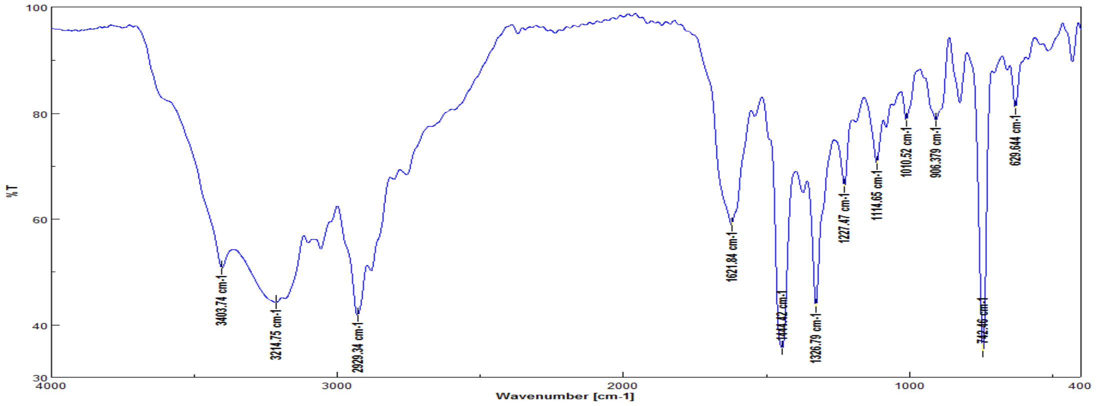

Fig.S13 IR spectrum of **2** (KBr disc)

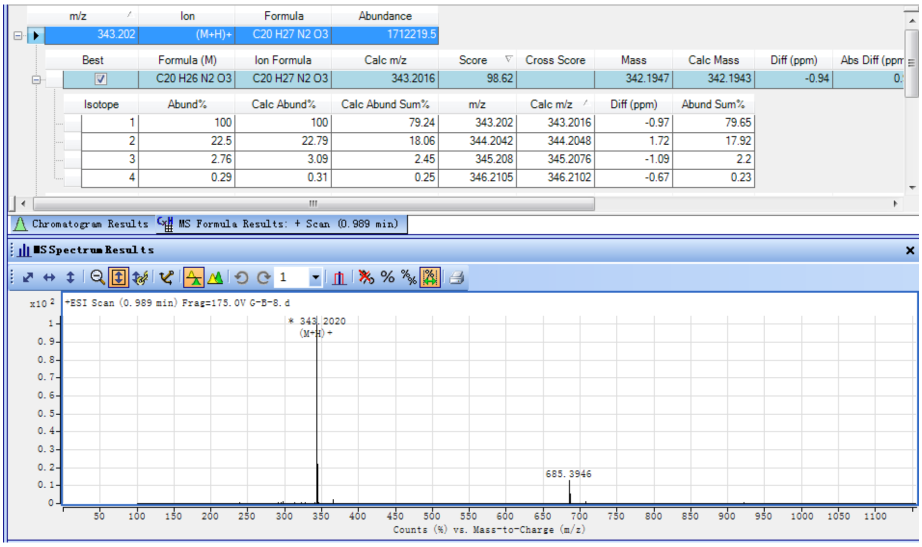

Fig.S14 HR-ESI-MS spectrum of **2**

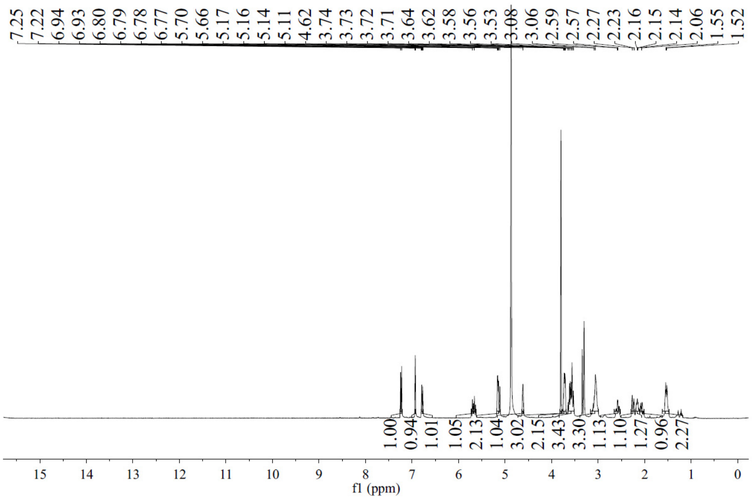

Fig.S15 ^{1}H NMR spectrum of **2** (600 MHz, CD_3OD)

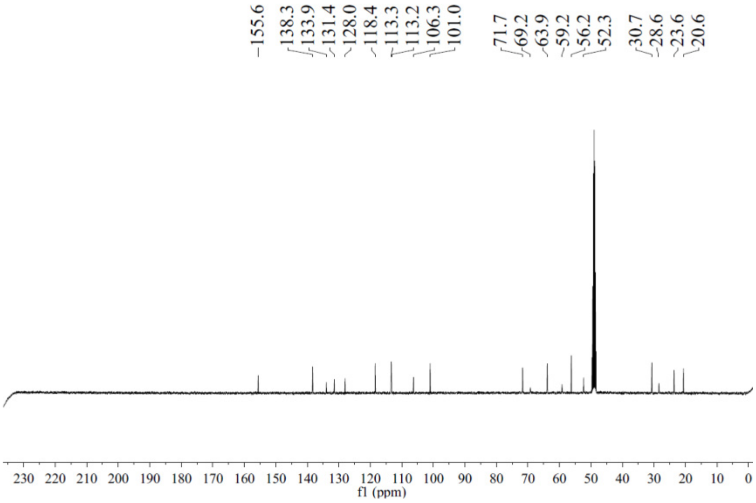

Fig.S16 ^{13}C NMR spectrum of **2** (150 MHz, CD_3OD)

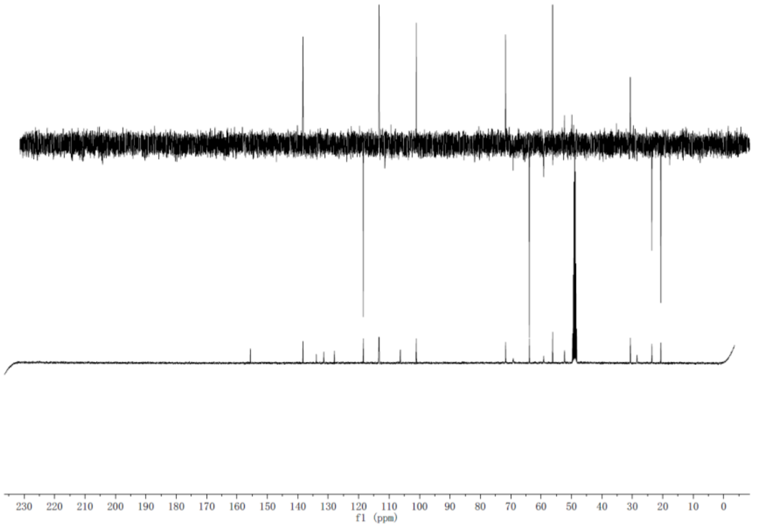

Fig.S17 DEPT-135 spectrum of **2** (150 MHz, CD_3OD)

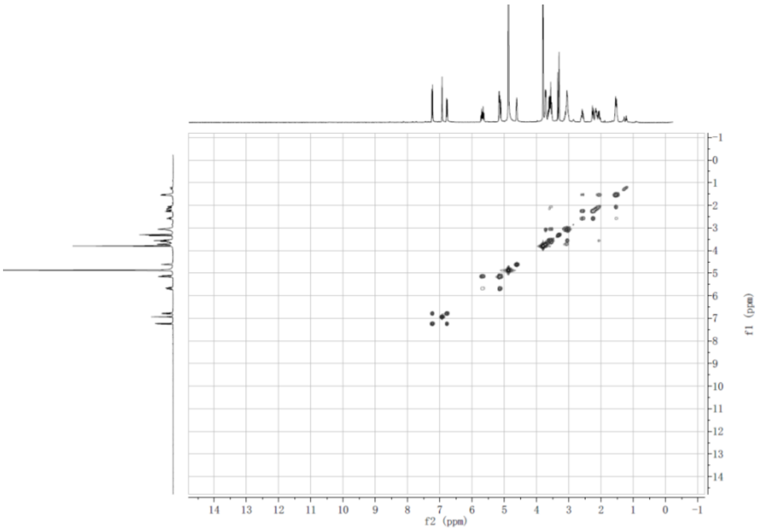

Fig.S18 ^{1}H-^{1}H COSY spectrum of **2** (CD_3OD)

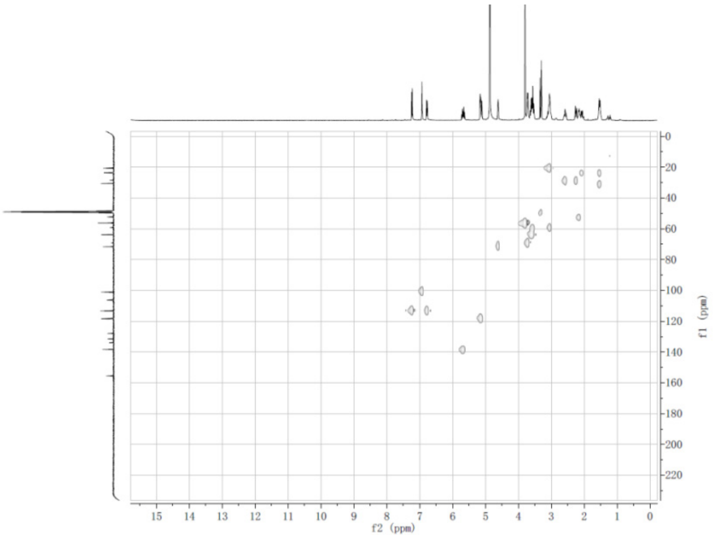

Fig.S19 HSQC spectrum of **2** (CD_3OD)

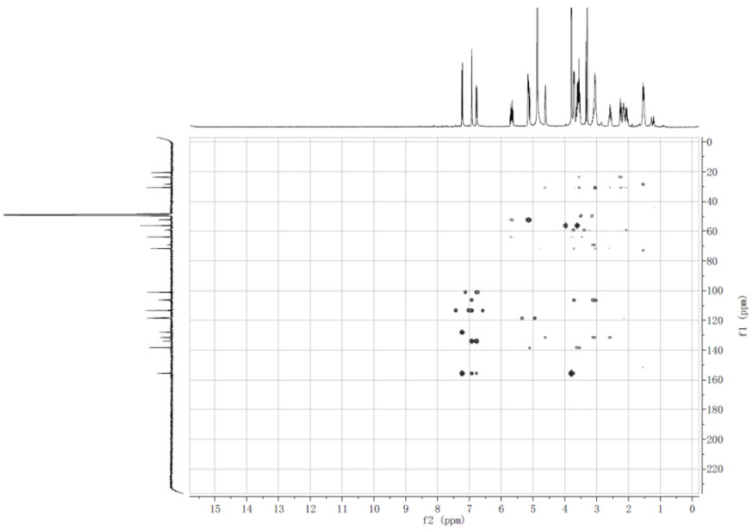

Fig.S20 HMBC spectrum of **2** (CD_3OD)

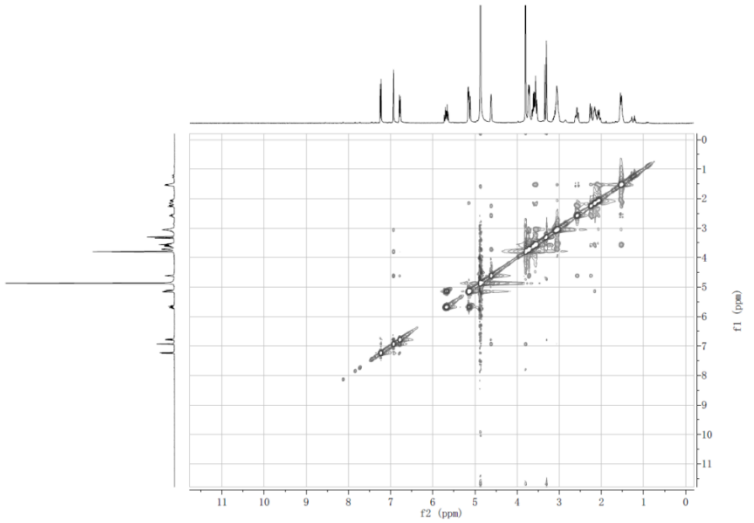

Fig.S21 NOESY spectrum of **2** (CD_3OD)

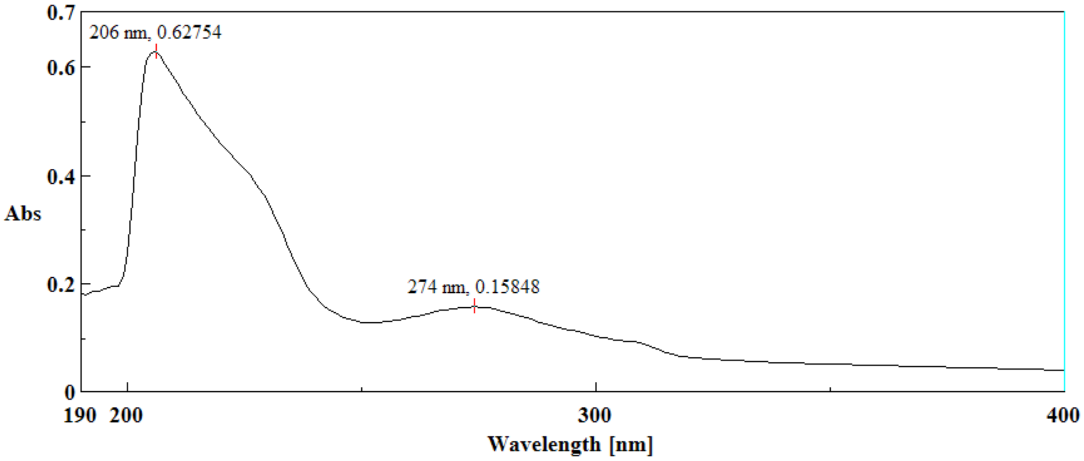

Fig.S22 UV spectrum of **3** (CH_3OH)

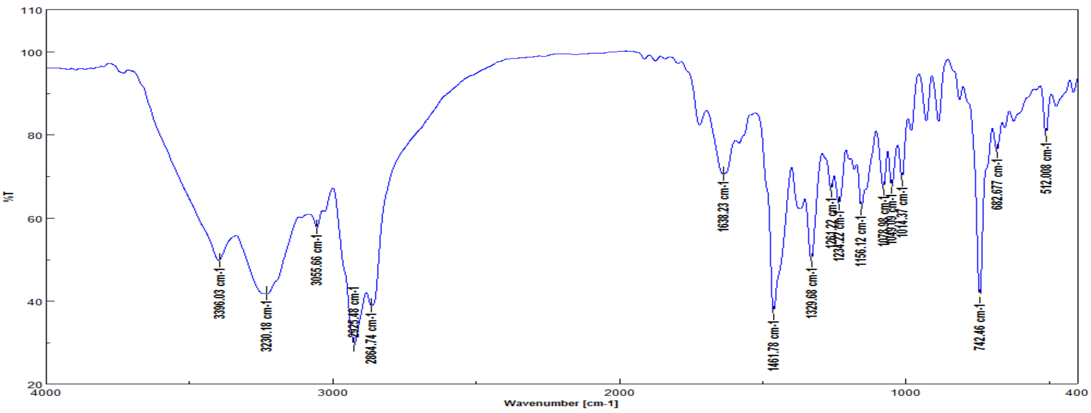

Fig.S23 IR spectrum of **3** (KBr disc)

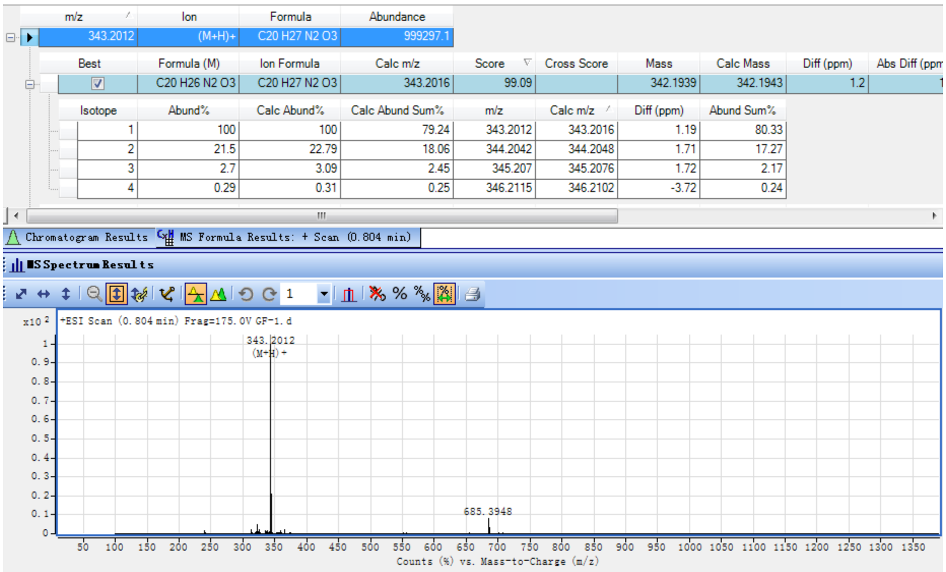

m/z	Ion	Formula	Abundance
343.2012	(M+H)+	C20 H27 N2 O3	999297.1

Best	Formula (M)	Ion Formula	Calc m/z	Score	Cross Score	Mass	Calc Mass	Diff (ppm)	Abs Diff (ppm
☑	C20 H26 N2 O3	C20 H27 N2 O3	343.2016	99.09		342.1939	342.1943	1.2	1

Isotope	Abund%	Calc Abund%	Calc Abund Sum%	m/z	Calc m/z	Diff (ppm)	Abund Sum%
1	100	100	79.24	343.2012	343.2016	1.19	80.33
2	21.5	22.79	18.06	344.2042	344.2048	1.71	17.27
3	2.7	3.09	2.45	345.207	345.2076	1.72	2.17
4	0.29	0.31	0.25	346.2115	346.2102	-3.72	0.24

Fig.S24 HR-ESI-MS spectrum of **3**

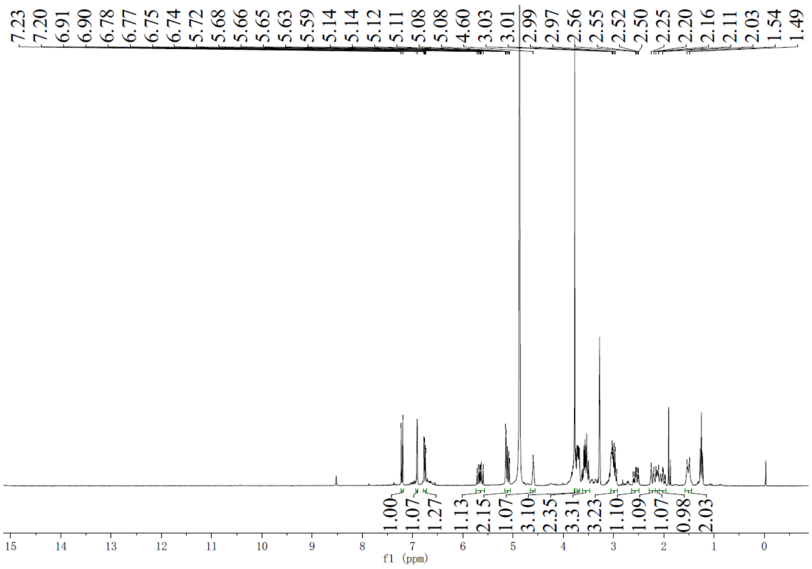

Fig.S25 ^{1}H NMR spectrum of **3** (400 MHz, CD_3OD)

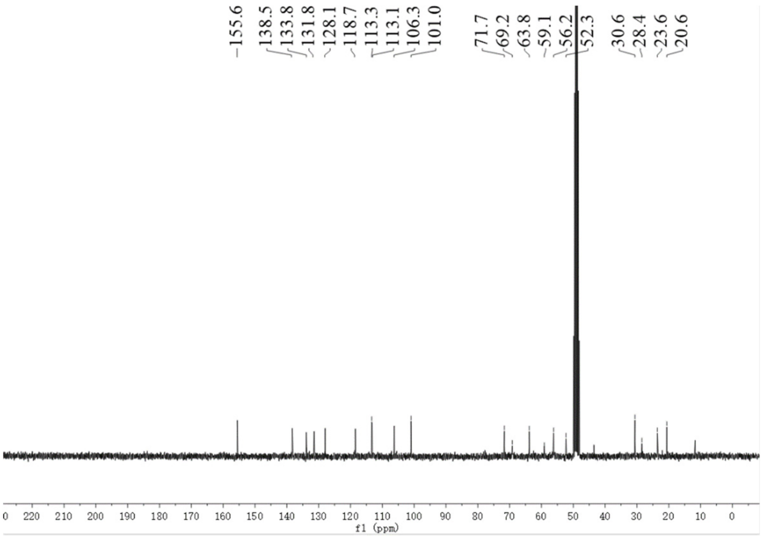

Fig.S26 ^{13}C NMR spectrum of **3** (100 MHz, CD_3OD)

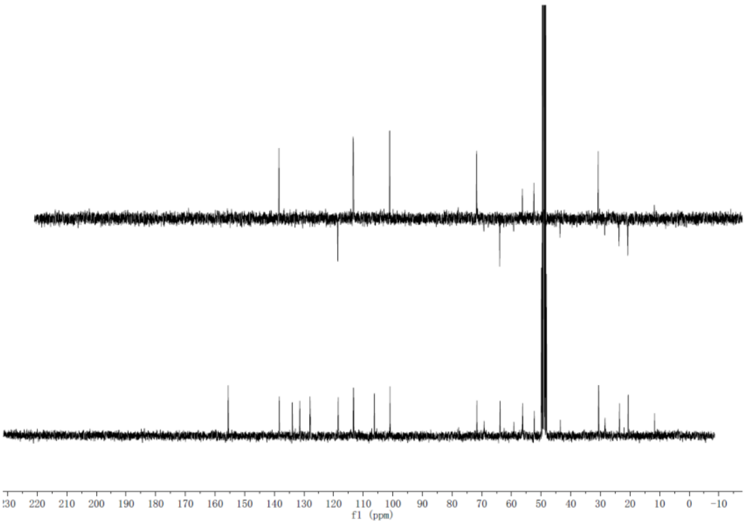

Fig.S27 DEPT-135 spectrum of **3** (100 MHz, CD_3OD)

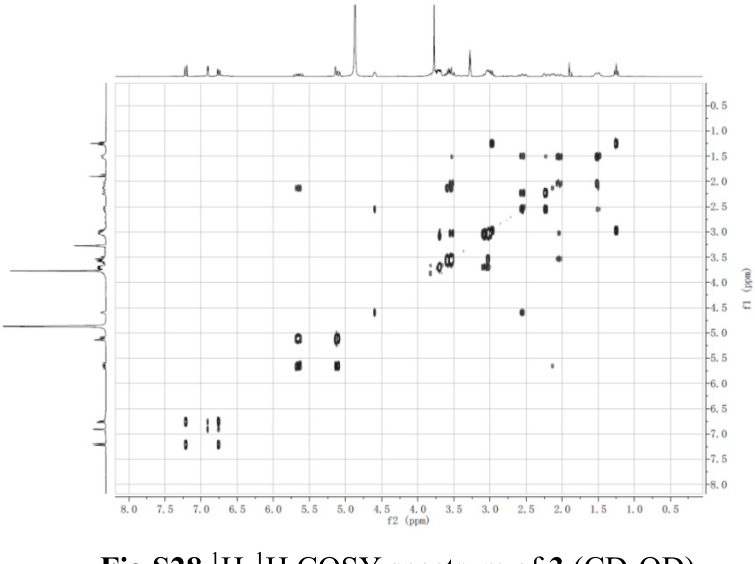

Fig.S28 ^{1}H-^{1}H COSY spectrum of **3** (CD_3OD)

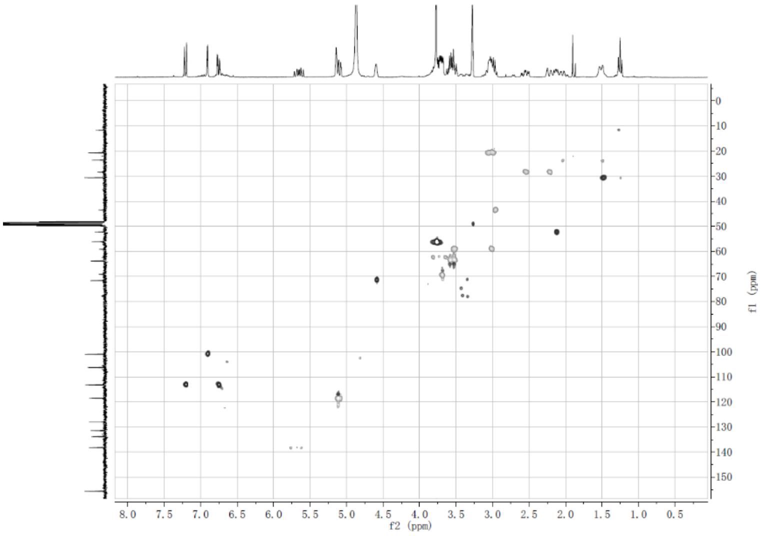

Fig.S29 HSQC spectrum of **3** (CD_3OD)

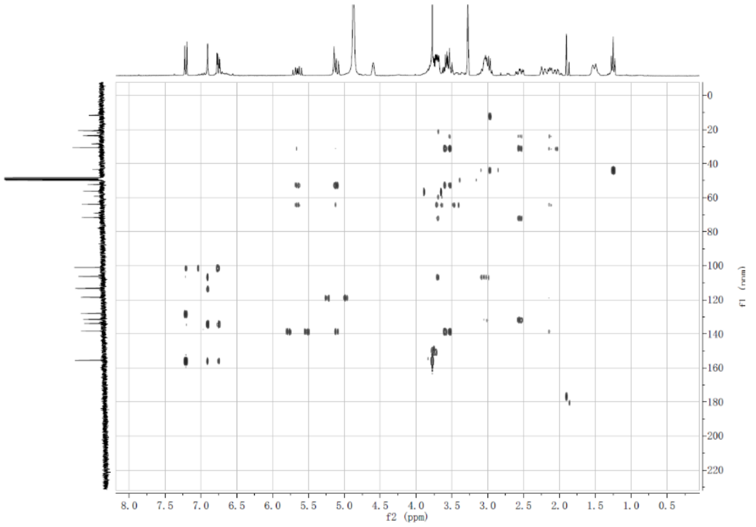

Fig.S30 HMBC spectrum of **3** (CD_3OD)

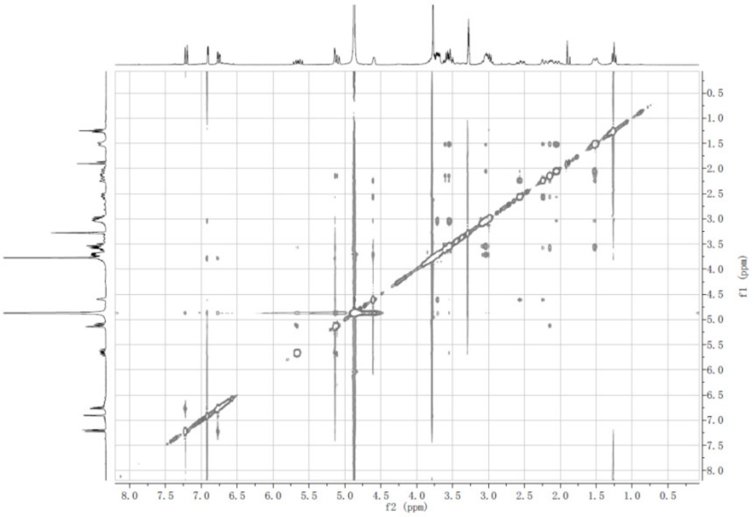

Fig.S31 NOESY spectrum of **3** (CD_3OD)
